# Salvage Radiation Therapy for Patients With Relapsing Glioblastoma Multiforme and the Role of Slow Fractionation

**DOI:** 10.3389/fonc.2020.577443

**Published:** 2020-12-08

**Authors:** I. Frank Ciernik, Yann Gager, Christof Renner, Sybille Spieker, Nicole Arndt, Karsten Neumann

**Affiliations:** ^1^ Department of Radiotherapy and Radiation Oncology, City Hospital, Dessau, Germany; ^2^ University of Zürich (MeF), Zürich, Switzerland; ^3^ PathoNext GmbH, Leipzig, Germany; ^4^ Department of Neurosurgery, City Hospital, Dessau, Germany; ^5^ Department of Neurology, City Hospital, Dessau, Germany; ^6^ General Pathology, Department of Pathology, City Hospital, Dessau, Germany; ^7^ Molecular Pathology, Department of Pathology, City Hospital, Dessau, Germany

**Keywords:** glioblastoma multiforme, GBM, glioma, radiotherapy, external beam radiotherapy, salvage, salvage therapy, reirradiation

## Abstract

**Background:**

Salvage radiation therapy (SRT) can be offered to patients with relapsing glioblastoma multiforme (GBM). Here we report our experience with a schedule extending the treatment time of SRT with the aim to prolong the cytotoxic effect of ionizing radiation while minimizing the cytotoxic hazards for the surrounding brain.

**Methods and Patients:**

From 2009 until 2017, 124 of 218 patients received radical resection, adjuvant chemo-radiation with photons and temozolomide (TMZ) followed by adjuvant TMZ. Re-irradiation was performed in 26 patients due to local relapse. Treatment schedules varied. Survival and molecular markers were assessed.

**Results:**

The median survival was respectively 12 months (9–14.5) of the 124 patients treated with tri-modal therapy and 19.2 months (14.9–24.6) for the 26 patients retreated with SRT (*p*=0.038). Patients who received daily fractions of 1,6 to 1,65 Gy to a total dose of >40 Gy had a median survival time of 24,6 months compared to patients treated with higher daily doses or a total dose of <40 Gy (p= 0.039), consistent with the observation that patients treated with 21–28 fractions had a median survival of 21,9 months compared to 15,8 months of patients who received 5–20 fractions (p=.0.05). Patients with Ki-67 expression of >30% seemed to perform better than patients with expression levels of ≤20% (*p*=0.03). MGMT methylation status, TERT promoter or ATRX mutations, overexpression of p53, p16, PD-L1, and EGFR were not prognostic.

**Conclusions:**

Re-irradiation of relapsing GBM is a highly valid treatment option. Our observation challenges hypofractionated stereotactic radiotherapy for retreatment and controlled trials on the fractionation dose for SRT are needed. Robust predictive molecular markers could be beneficial in the selection of patients for SRT.

## Introduction

Glioblastoma multiforme (GBM) is an aggressive form of brain tumor. Classical local therapies such as surgery and radiotherapy have complemented with chemotherapy and electric treatment fields after initial multimodal therapy (1–3). The cure rates however remain disappointing and dealing with local relapse is part of clinical routine (4). Thus, second line or salvage therapies are used in many patients including re-operation, re-irradiation or a combination of both modalities. In recent years, systemic therapies with cytotoxic agents or targeting molecules have been advocated for salvage therapy, eventually within a clinical trial often preferred (5). However, if it comes to SRT, several factors influence the choice of the appropriate radiotherapy technique, such as the dose and location of prior radiotherapy, the cumulative doses and the risk of damage to healthy brain tissue or radionecrosis. The size and gross tumor volume (GTV) of the relapsing or persisting tumor impact on fractionation schedules and techniques. If combined modality is chosen, the retreatment fields might be minimized to the GTV. Any known technique of photon-based external beam therapy such as gamma knife, robotic radiosurgery, radiosurgery on linear accelerator-based radiotherapy have been reported to be suitable for SRT, including a wide range of fractionation schedules using single fraction SRT, highly or moderate hypo-fractionated of conventionally fractionated schedules yielding acceptable clinical results. During the last decades, time-saving schedules implementing short treatment series has become popular due to the palliative character of SRT and to minimize the treatment burden for the patients (6–12).

In the present study, we review our experience with patients treated with SRT in respect of survival and potential biological markers associated with prolonged survival.

## Methods and Materials

### Study Design and Patients

This research was a retrospective analysis of a cohort of all consecutive patients who underwent SRT at a single tertiary health care facility from January 2009 until December 2017, prior to introduction of systemic therapy of GBM patients with electric treatment fields. A total of 218 patients were diagnosed with primary GBM at the City Hospital of Dessau, Germany and 124 patients were treated with trimodal therapy consisting of radical total resection, postoperative adjuvant radiotherapy to 60 Gy with concomitant TMZ followed by adjuvant TMZ. The minimal follow-up for all patients was 2 years. All patients consigned to analysis and communication of their data in anonymized reports or publications, and the case review study was approved by the ethics’ committee [Ethikkommission der Ärztekammer Sachsen-Anhalt, Halle (Saale), Germany] and internal review board.

### Surgery

Surgery was planned on the basis of the preoperative magnetic imaging (MRI) studies. After trepanation and visualization of the tumor, guidance included intraoperative ultrasound, neurostimulation and fluorescence (13). Complete surgical aspiration was defined by a post-surgical MRI the day after surgery.

### Chemotherapy

Initial chemotherapy consisted of concomitant temozolomide (TMZ) during primary radiotherapy at a dose of 75mg/m^2^, and all 26 patients received TMZ during primary RT. After the end of chemoradiation, adjuvant TMZ with a first course of 150 mg/m2 (days 1 to 5) followed by more courses with 200 mg/m^2^ (days 1 to 5) was given to all patients. Courses were repeated every 28 days to at least six cycles or until relapse was observed.

### Radiotherapy

Primary chemo-radiation was performed with 6 MV photons using coplanar or non-coplanar beam geometries of dynamic or static modulated fields in the vast majority of patients. All patients received 1,8 to 2,0 Gy daily to a total dose of 59,4 to 61,4 Gy. Patients treated subsequently with SRT had a Karnofsky performance status of >70%. All patients received 3D-treatment planning on a planning computer tomography (CT HiSpeedFXI, GE Solingen, Germany) or on a Toshiba Aquillon LB (Canon Medical Syst. Neuss, Germany). All patients had magnetic resonance imaging studies available for target volume definition and SRT planning. The planning CT (Philips, Eindhoven, Netherlands) was fused with the MRI (Philips Achieva 3.0.T Hamburg, Germany) using the T1 imaging with Gadolinium and the T2-imaging studies to define the gross tumor volume (GTV) of the relapse (Eclipse, Varian, Palo Alto, CA, USA). A margin of 2–4 mm surrounding the GTV was used to define the primary planning target volume disregarding the perilesional edema. However, the PTV varied according to the physician’s discretion. The PTV was often asymmetrically extended into the direction of the neighboring surface of the resection cavity to a maximum up to 6 mm, or towards parts of edematous structures or suspected areas of tumor cell infiltration. The planning was performed with ARIA version 9.0 and 11.0 after 2012 (Eclipse, Varian, Palo Alto, CA, USA). The treatment was 3D-conformal in twelve cases and highly conformal using IMRT, VMAT or combined techniques in 14 patients. The choice for the used technique was the result of comparative planning and the dose to organs at risk in the sum plans with the primary radiotherapy defined the use of the beam geometry for SRT. The average number of fractions used was 21,3 (range: 5–28). To have a comparable number of patients in groups analyzed in respect of treatment duration, a cutoff of four treatment weeks was chosen. Fourteen patients were treated with 5–20 fractions and 12 patients with 21 to 28 fractions.

### Histology and Molecular Analysis

Tissue samples from GBM where fixed using 4% phosphate buffered formaldehyde and embedded in paraffin according to standard procedures. H&E staining and immunohistochemistry (IHC) were performed on 3 µm paraffin sections. Expression of three protein markers (Ki-67, p16, PD-L1) and *EGFR* gene amplification were analyzed on a subset of 22 tumor cases. The markers were chosen from a larger molecular panel including *MGMT* promotor methylation, *IDH1/2* mutation, *BRAF* (V600E) mutation, 1p19q-deletion, *TERT* promotor mutation as well as the IHC-markers (ATRX, CD44, EGFRvIII, TP53 etc.) routinely applied in our molecular GBM testing. Corresponding data are not shown. The four markers selected proved to be the most promising ones due to their high levels of variance and the limited number of cases available for analysis.

For IHC the following antibodies were used: Ki-67 (Dako), p16 (Cintec/Roche, Basel Switzerland) and PD-L1 (22C3, Dako). Staining was performed with the Dako Autostainer Link 48 using protocols supplied by the manufacturers: Ki-67 (ready-to-use solution, demasking pH 6, FLEX rtu detection), p16 (ready-to-use solution, demasking pH9, FLEX rtu detection), PD-L1 (1:50 dilution, demasking pH6, FLEX+mouse+DAB enhancer).

Staining results were examined by experienced pathologists under the microscope. Expression was quantified as the proportion of stained tumor cells in percent. The PD-L1 scoring is therefore equivalent to the tumor proportion score (TPS) applied to other tumor types (14). A hundred tumor cells were counted in every case.

*EGFR* gene amplification was evaluated by FISH using the ZytoLight SPEC EGFR/CEN 7 Dual Color probe (Zytomed, Berlin, Germany) according to the supplied protocol. *EGFR* amplification was scored after examination of 100 tumor cells as follows: EGFR/CEN7-ratio <2,0 (no amplification), EGFR/CEN7-Ratio ≥2,0 (amplification) (15, 16).

### Statistical Analysis

Statistical analyses were done in R (17, 18) with the packages ggplot2 and survminer (19). Survival data was plotted as Kaplan-Meier (KM) curves with median survival. We used the log rank test to obtain a p-value and compare statistically two or three groups. We calculated the median as well as the 0.95 low confidence limit (LCL) and 0.95 Upper Confidence Limit (UCL). Furthermore, we calculated the beta coefficient using Cox proportional hazard model to investigate the influence of different numeric factors on survival. Mann U Whitney test was used for non-parametric comparison. Multivariate analysis was omitted due to the size of the cohort.

## Results

**Figure 1:**
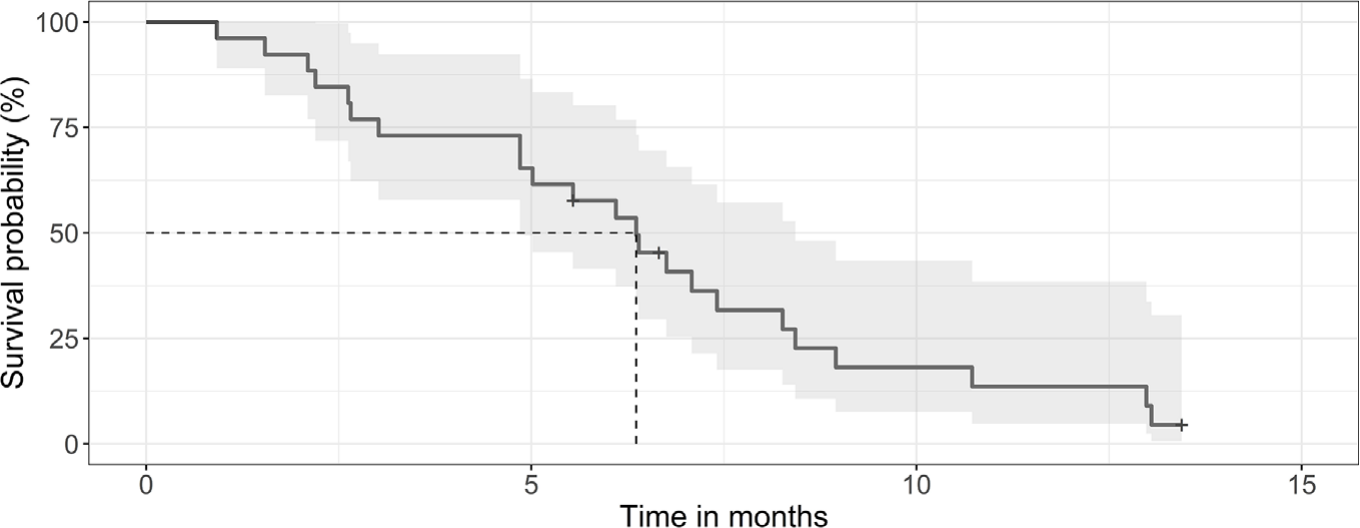
Kaplan-Meier curve for survival of patients after SRT. The median survival was 6.4 months (0.95 LCL–0.95 UCL: 4.8–8.4 months) for the 26 patients after the end or SRT.

**Figure 2:**
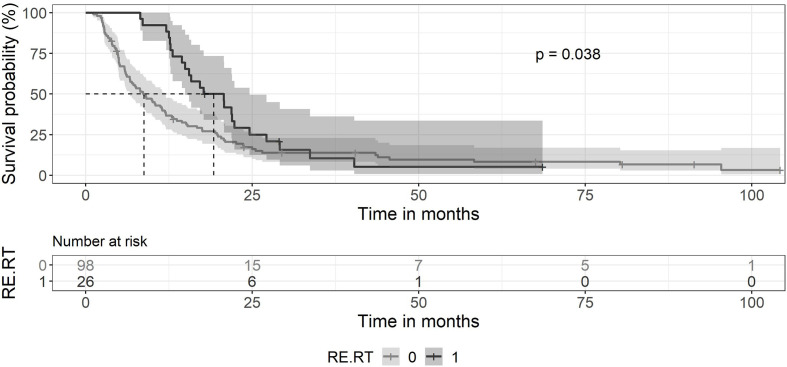
Kaplan-Meier curves for survival between patients treated with primary chemoradiation without SRT and patients retreated with SRT. Ninety-eight patients without SRT (gray line) showed a median survival of 8.8 months (0.95 LCL–0.95 UCL: 6.7–11.9 months). The median survival of patients with SRT (black line) was 19.2 months (0.95 LCL–0.95 UCL: 14.9–24.6 months).

The median survival of the 124 patients treated primarily with trimodal therapy was 12 months (0.95LCL–0.95UCL: 9–14.5 months). The median survival of the patients treated with SRT was 19.2 months (0.95LCL–0.95UCL: 14.9–24.6 months) and 6,4 months (0.95LCL–0.95UCL: 4.8–8.4) after the end of the primary radiotherapy (**Figure 1**). For the subsets of patients without SRT (n = 98), the median survival after surgery was 8.8 months (0.95LCL–0.95UCL: 6.7–11.9 months). Patients treated with SRT survived longer (p = 0.038) (**Figure 2**).

### Age

The average age of the entire group treated with trimodal therapy was 67.4 years (range: 26–94 years). Patients who received a second series of radiotherapy after primary radiochemotherapy had an average age of 63.6 years (range: 25–81) (p>0.05). Patients offered SRT were selected on performance and the presentation of a single lesion accessible to SRT. Results of the Cox Proportional-Hazards model testing for age give a positive (0.03983) and a highly significant beta coefficient (0.0291), suggesting age as a major risk factor for shortened survival after SRT.

### GTV and PTV

**Figure 3:**
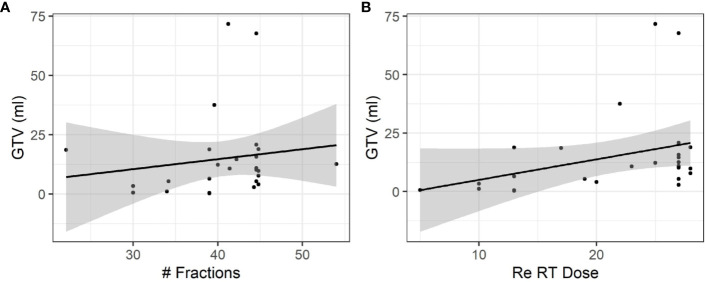
Influence of the size of relapsing GBM on fractionation and total dose. **(A)** A moderate positive association was observed between the size of relapsing GBM and the total number of fractions (r = 0.35). **(B)** A weak positive association was observed between the size of relapsing GBM and the dose (r = 0.15).

The mean GTV was 15.0 ml (range 0.3–71.7 ml) and the mean PTV was 66.8 ml (range 2.9–185.5 ml). Larger GTV or PTV would more likely be treated with prolonged treatment courses with more than 20 fractions. Correlations between GTV and number of fractions (r = 0.35) and correlations between GTV and Re RT Dose (r = 0.15) (**Figure 3**).

Patients with increasing GTV or PTV had poorer, however still similar survival rates: results of the Cox Proportional-Hazards model for GTV give a positive (0.013) but no significant beta coefficient (0.236), and the PTV gave a positive (0.001) but not significant beta coefficient (0.773).

To test, whether the coverage of the GTV could impact on the survival, the ratio of PTV/GTV was analyzed. PTV/GTV ratio gave a negative (-0.0015) but not significant beta coefficient (p = 0.884). Thus, an increasing PTV/GTV ratio was not associated with enhanced survival rates.

### Radiotherapy-Free Time (Days)

SRT was performed on an average of 338 days (SD 265.5; range 68–1188 days) after the end of chemoradiation. The Cox Proportional-Hazards model for the variable radiotherapy-free time (days) gave a negative (-0.009316) and highly significant beta coefficient (4.98e-05). Postponing SRT to 4–13 months after the end of primary radiotherapy was associated with prolonged survival (p < 0.05). Thus, survival time after initial surgery correlated with the radiotherapy-free time.

### Influence of the Fractionation Schedule on Survival

**Figure 4:**
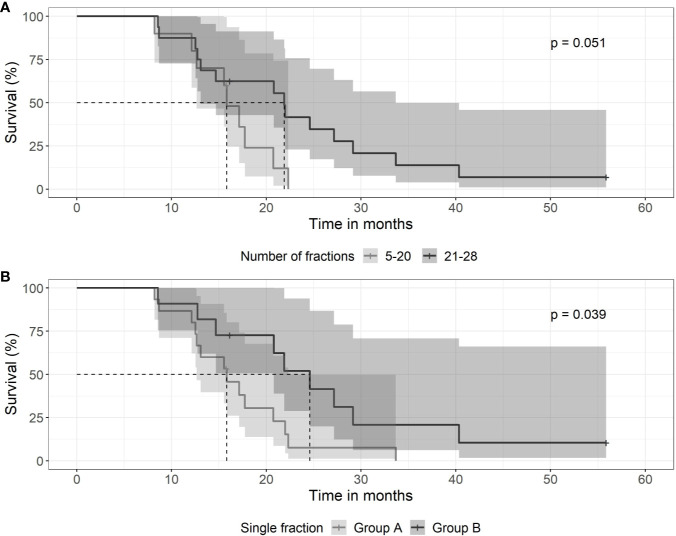
Kaplan-Meier curves for survival between groups of patients with differing fractionation schedule. **(A)** SRT and number of daily fractions. Patients treated with 5–20 fractions (gray line) had a median survival time of 15.8 months (0.95 LCL–0.95 UCL: 12.7–NA months), compared to patients treated with 21–28 fractions (black line) with a median survival time of 21.9 months (0.95 LCL–0.95 UCL: 13.1–33.7 months). **(B)** Patients treated with low daily doses of 1,6 to 1,65 Gy to a total dose exceeding 40 Gy (black line) had a median survival time of 24.6 months (0.95 LCL–0.95 UCL: 20.8–NA months), compared to those patients treated to a dose of <40 Gy (gray line) with a median survival time of 15.8 months (0.95 LCL–0.95 UCL: 12.7–22.3 months).

Fractionation schedules used were heterogeneous. Patients were treated with daily doses of 1,6 to 1,65 Gy or with 1,8 to 2,0 Gy. For hypofractionated radiotherapy, daily doses of 3 or 6 Gy were used. Patients treated with a total number of ≤20 treatments had a median survival time of 15.8 months (0.95 LCL–0.95 UCL: 12.7–NA months), compared to patients treated with 21–28 fractions with a median survival time of 21.9 months (0.95 LCL–0.95 UCL: 13.1–33.7 months) (p = 0.051) as shown in **Figure 4A**. Patients treated with daily doses of 1,6 to 1,65 Gy to a total dose >40Gy had a median survival time of 24.6 months (0.95 LCL–0.95 UCL: 20.8–NA months), compared to those patients treated to a dose of ≤40Gy with a median survival time of 15.8 months (0.95 LCL–0.95 UCL: 12.7–22.3 months) (p = 0.039) as shown in **Figure 4B**.

### Ki-67-, p16-, PD-L1-IHC, and *EGFR* Amplification

**Figure 5:**
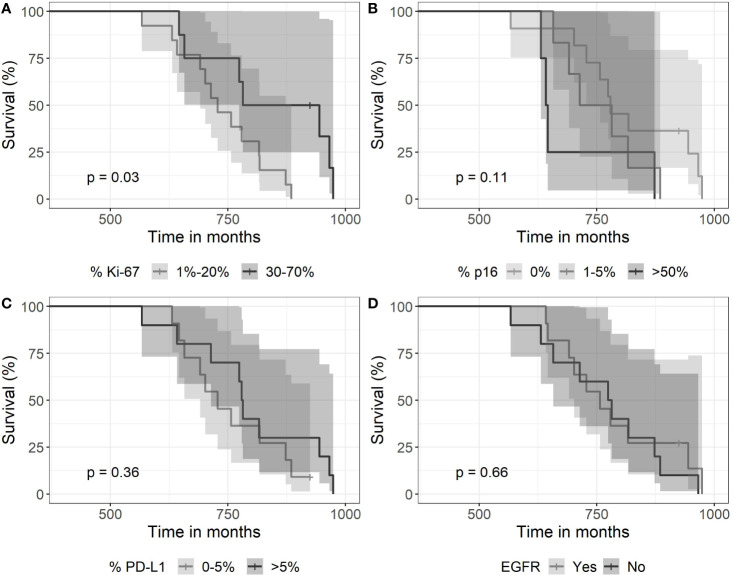
Kaplan-Meier curves for survival as a function of molecular markers. **(A)** Expression of >30% of Ki67—associated with longer survival (p=0.03). **(B)** The expression of p16 did not associate with survival (log-rank test with a p-value of 0.11), **(C)** neither did a high expression of PDL-1 (p=0.36), nor **(D)** EGFR (immunohistochemistry) expression (p=0.66).

Ki-67 expression proved highly variable among the 22 tumor cases. It ranged from about 1% to 70%. Ki-67 showed a high difference in survival distributions (p = 0.03). Survival was lower for patients with an expression of 1%–20% of Ki-67 in comparison to patients with an expression level of 30%–70% (**Figure 5**). Fourteen patients with a Ki-67 expression of 0%–20% received SRT with 13–28 fractions over a period of 39–64 days, whereas patients with high Ki-67 expression of 30%–70% were treated with 15–28 fractions over 13–49 days. Thus, there is no association of Ki-67 expression with treatment length shown in **Figure 4**.

In contrast, the other two expression parameters p16 and PD-L1 did not show high difference in survival distribution between the different categories. A complete loss of p16 expression was observed in 11 cases. High expression >50% was noticed in 5 cases. PD-L1 expression proved positive in 15 tumors with scores between 1% and >50%. Eleven tumors had *EGFR* gene amplification but did not associate with survival time.

## Discussion

A variety of salvage options exists for relapsing GBM including re-operation, systemic and cytotoxic therapies or a combination of treatment modalities (20). The choice of salvage therapy depends on the circumstances and clinical fitness of the patient. SRT has been already discussed more than 30 years ago for patients suffering from relapsing GBM (21). Re-irradiation bears a burden for patients’ time and management, and cumulative toxicity to healthy brain tissue. However, with the improvement of radiotherapy techniques and computing in the field of stereotactic and hypofractionated radiotherapy or radiosurgery, SRT has been increasingly accepted as a valuable salvage treatment alone or in combination with other modalities (22).

The patients selected for SRT in the present series survived almost 18 months after initial therapy. Three survived more than 2 years and aiming a survival time of 2 years for good candidates for SRT seems realistic in general practice outside controlled studies (cf. **Figure 1**). This observation is consistent with previous reports of patients treated with SRT who experienced a prolonged survival compared with patient without SRT (23). In respect of the timing of SRT, we observed, that late use of SRT was associated with better survival, consistent with the observations of Zemlin *et al.* who suggest that early SRT should be beneficial preferentially in the context of previous salvage surgery (24). Therefore, the knowledge to better define and select patients with prolonged progression of disease and optimal strategies to incorporate SRT in the treatment portfolio seem mandatory.

Age has been a risk factor for worse outcome in our series of SRT, constant with our population-based analysis, that young age is a major factor for long-term survival (25). It is therefore especially tempting to use short treatment courses in the elderly to minimize the time burden of medical care. From studies that indicate equipotency of hypofractionated radiotherapy in the elderly, we assume that age is likely to be a stronger predictor than dose and fractionation schedules, especially for candidates for SRT. Nevertheless, we agree with other authors that age should not be a determining criterion to withheld SRT (26).

In the present series, in the absence of randomized and controlled trials of salvage radiotherapy, we took an alternative approach and we sought to extend the treatment time and to minimize the daily dose of cytotoxic ionizing radiation. The rationale behind this is that GBM represent a highly heterogeneous population of tumor cells with considerable ability of redistribution and regrowth of dormant or hypoxic cells. Therefore, a “mild” but prolonged regimen might be suitable, taking into account the low alpha/beta ratio of the brain. As shown in **Figure 4**, “mild” re-irradiation with daily doses of 1,6–1,65 Gy (e.g. 28 x 1,65 Gy) seems to be potentially advantageous over shorter regimens. Indeed, Rasmussen *et al.* investigated reduced dose rate to deliver standard doses of daily 2 Gy to reduce potential toxicity to neighboring brain tissue have tested the feasibility of a “mild” radiotherapy (27). Therefore, the optimal fractionation schedules for SRT remain an important aspect for prospective and controlled trials. Controlled and randomized trials investigating different fractionation schedules are important and may show improved outcome if adjusted for age, while remembering that accelerated radiotherapy seems to yield comparable results as standard fractionation in the elderly regarding progression-free survival but not overall survival (28).

The role of biological markers for GBM remains controversial except for *MGMT* and *IDH1/2* for primary GBM (29). *MGMT* did not associate with prognosis in the present small cohort study. The number of markers tested here leaves the possibility open that the association of Ki-67 with prognosis happened by chance, although Ki-67 has been discussed previously and its prognostic value for primary GBM suggested by earlier studies (30). Ki-67 has been associated with improved prognosis in primary GBM in an early report (31), confirmed by others who reported a positive correlation of Ki-67 and survival in a series of primary GBM (32). Thus, our observation from this small series is consistent with others.

The main weakness of our study is its retrospective design analyzing a small cohort generated over almost a decade in a single institution. Case series are important, although conclusions are preliminary, and confirmation of the results are important, either by other institutions or with controlled studies. Treatment allocation to short or long courses were not entirely clear, and certainly biased by the patient’s performance status, clinical assessment, or patients’ preferences. The location and the size of the tumor amendable to retreatment with sole involvement of one hemisphere represent another therapy bias. Recruiting patients into a trial on PET-based RT planning using a short fractionation schedule influenced the allocation to extended radiotherapy courses, as well (33).

## Conclusions

In our hands, radiotherapy seems to be a useful salvage treatment option for relapsing GBM in selected patients. The optimal fractionation schedule, the total dose as well as the optimal beam geometry to be used remain largely unknown. Our data allow us to hypothesize, that extending the total treatment time by means of reducing the daily dose of SRT could be a sensible way and confirmation in a controlled setting is warranted.

## Data Availability Statement

The raw data supporting the conclusions of this article will be made available by the authors, without undue reservation.

## Ethics Statement

The studies involving human participants were reviewed and approved by Ethikkommission der Ärztekammer Sachsen-Anhalt, Halle(Saale), Germany; IRB, Städtisches Klinikum Dessau, Dessau, Germany. Patients agreed to handling and analysis of their anonymized or pseudonymized personal data, most of all outcome analysis within the frameworks of quality assurance of medical care as a part of hospital services.

## Author Contributions

IC, YG, CR, SS, and KN designed the study. NA was in charge of the histology analysis. NA and KN were in charge of the molecular analysis. IC, KN, and NA analyzed the data. YG was in charge of the statistics. IC, YG, CR, SS, and KN wrote the paper. IC, SS, CR, and KN edited the paper. All authors contributed to the article and approved the submitted version.

## Conflict of Interest

Author YG was employed by PathoNext GmbH.

The remaining authors declare that the research was conducted in the absence of any commercial or financial relationships that could be construed as a potential conflict of interest.
